# Rural-urban disparity in uptaking skilled antenatal care visits by pregnant women in Bangladesh: Zero and One Inflated Poisson regression model

**DOI:** 10.1371/journal.pone.0318341

**Published:** 2025-01-29

**Authors:** Lubana Tanvia, M. Ershadul Haque, Wasimul Bari

**Affiliations:** Department of Statistics, University of Dhaka, Dhaka, Bangladesh; Jahangirnagar University, BANGLADESH

## Abstract

**Background:**

Utilization of maternal health care services, specifically, antenatal care services from skilled health providers have been given utmost priority in low- and middle-income countries over years with a view of mitigating complications during pregnancy as well as safeguarding the health and survival of both mother and newborn. However, there is a general tendency of pregnant mothers in Bangladesh of receiving skilled antenatal care (SANC) service once, or even never which refrains us to ensure World Health Organization (WHO) recommended eight plus SANC visits, additionally, to meet Sustainable Development Goal (SDG) number three.

**Objectives:**

The study aims at assessing how the average number of SANC visits taken by the reproductive women in Bangladesh changes over the time in rural and urban areas together with finding out the potential demographic and socio-economic factors associated with SANC visits by addressing possible accumulation of zero and one counts in SANC visits.

**Methods:**

In this study, data have been retrieved from last four waves of Bangladesh Demographic and Health Surveys ranging from 2011 to 2022 and later combined together to form a pooled dataset. Non-parametric Kruskal-Wallis test has been performed for exploring unadjusted association of covariates with the response and Mann-Whitney U test has been conducted for multiple comparison in case of significant association for a covariate having more than two categories. For checking the existence of concurrent inflation at zero and one in the pooled dataset, partial score test has been performed. Based on the results of score test, Zero and One Inflated Poisson regression model has been fitted to the pooled dataset, where an interaction term between area of residence and survey year has been considered for trend analysis.

**Results:**

The study highlights that the rate of mean SANC visits is rapidly rising with time among pregnant women not only from the urban areas but also from the rural areas of Bangladesh. However, the rate of change in uptaking the SANC visits is higher in rural area compared to urban area. It was observed that for a given survey year, the rate of mean SANC visits was higher among women from urban areas compared to that among women from rural areas of Bangladesh. This study provides the evidence of 17.2% extra zero counts and 1.1% extra one counts in the pooled dataset.

**Conclusion:**

The study results depict that inequity in SANC services based on area of residence prevails in Bangladesh. However, the gap in the rate of mean SANC visits in rural areas compared to urban areas has gradually reduced over time. Based on the results, the study comes up with some recommendations to facilitate the policy makers in improvising strategies and ensuring sustainable rise in SANC counts as well as WHO recommended positive pregnancy experience in the country to meet SDGs.

## Introduction

It is apparent from the evidence of low- and middle-income countries that high-risk pregnancies can cause death of the newborn as well as the mother at and shortly after the time of birth [1]. According to the United Nations Inter-agency Group Child Mortality Estimation (UN IGME), globally the estimate of deaths among children within the first 28 days of life was 17 deaths per 1000 live births in 2022, i.e., around 2.3 million neonates died globally which covered 46.94% of under-five children deaths that occurred in 2022 [2]. Meanwhile in 2020, the estimate for maternal death was 287000 globally and approximately 9.8% deaths of reproductive mothers were due to maternal health complications according to United Nations Maternal Mortality Estimation Inter-agency Group (MMEIG) [3]. Bangladesh is one such country with high neonatal mortality rate (NMR) and maternal mortality ratio (MMR). Although over the decades both NMR and MMR in Bangladesh have decreased to a great extent, such as NMR has decreased from 48 per 1000 live births in 1993–94 to 20 per 1000 live births in 2022 [4] and MMR has reduced from 318 per 100000 live births in 1993–94 to 123 per 100000 live births in 2020 [5], more attention is still needed in this aspect as we are lagging behind to reach the targets of the SDG number 3 (Good Health and Well-being) and the 4^th^ Health, Population and Nutrition Sector Program (HPNSP). Among several strategies taken to minimize the NMR and MMR in Bangladesh, antenatal care (ANC), the care given during pregnancy including medical interventions to safeguard the health of both mother and fetus, have emerged to be one of the crucial factors [6].

In conjunction with the SDGs (3.1) and (3.2) which aim at declining MMR to 70 per 100000 live births and NMR to 12 per 1000 live births by the year 2030, respectively [7], WHO recommends to prioritize the quality as well as the quantity of ANC visits and suggests a minimum of eight ANC visits under the supervision of skilled health attendants in the “2016 WHO ANC model” [8]. According to WHO, skilled health attendants refer to the health providers such as qualified doctors, nurses and midwives trained to conduct normal pregnancies and provide care to mothers and newborn before and during delivery [9] and skilled antenatal care (SANC) visits should incorporate obstetrical examination for ensuring timely care-seeking during emergencies, advising and educating mothers about possible health complications as well as counseling them on nutrition, healthy lifestyle, safe and healthy birth and maternal and newborn care [8].

Although an increasing pattern is noticeable in four plus ANC visits in Bangladesh over the decades, from 6% in 1993–94 to 46% in 2017–18, then a reduction to 41% in 2022 [4], access to SANC service and its utilization are still needed to be prioritized in mainstream health systems to meet SGDs and WHO recommended positive pregnancy outcome in Bangladesh. Hence analysis of SANC visits is of utmost importance in Bangladesh.

Several research works have been conducted to identify potential determinants that influence ANC visits in Bangladesh, which emphasized on WHO guideline of taking at least four ANC visits. Mohammad et al. in their study used a sequential logistic regression model and concluded that interaction between place of residence and maternal education plays a significant role in increasing complete ANC visits [10]. Khan et al. used Bayesian logistic regression model in their study to determine the effect of unwanted pregnancy on SANC visits in Bangladesh [11]. In the studies mentioned above, ANC visits had been taken as binary response instead of count response variable. Islam and Masud found that some demographic and socio-economic variables play pivotal role in determining the number of ANC visits [12], meanwhile, another study conducted by Islam et al. obtained similar results [13]. A negative binomial regression model was used for analysis purposes in both studies. Hossain et al. used zero-truncated negative binomial model and Haque et al. fitted a zero-truncated Poisson model to investigate the potential determinants for the adequate number of ANC visits by women in Bangladesh [14, 15]. However, in practice, in developing countries like Bangladesh, one may witness large frequency of zeros in ANC counts causing over-dispersion and application of Poisson regression model or negative binomial regression model in such zero-inflated count data may give inconsistent and less precise estimates of the parameters. Several classes of mixture models have been proposed to handle count data that account for excessive zeros. Ahmed and Mallick used zero modified count regression models for analyzing ANC counts in Bangladesh [6].

In many situations, besides excess zero counts, accumulation of excess one counts may be evident in the dataset [16–18]. The application of Zero Inflated Poisson (ZIP) model in such situation might provide misleading results due to underestimation of one counts [16]. For dealing with such zero and one inflated data, Melkersson and Olsson proposed Zero and One Inflated Poisson (ZOIP) distribution with an application of ZOIP model in dental epidemiology [19].

In most of the studies in literature, ANC service received from any type of providers had been analyzed for finding the potential determinants. In this study, an attempt has been made to explore the pattern of skilled antenatal care (SANC) visits taken by the reproductive women in Bangladesh over the time. It is also of interest to find out the potential factors associated with SANC visits by using an appropriate regression model so that the results obtained from this analysis may provide some recommendations to meet SDGs (3.1) and (3.2).

For this purpose, data have been extracted from four consecutive BDHSs conducted in 2011, 2014, 2017–18 and 2022 and later combined together to create the final dataset. The observations obtained on the SANC of this dataset may be zero and one inflated because (i) most pregnant women in Bangladesh do not take SANC visits at all causing inflation at zero and (ii) there is a general tendency among the majority of pregnant women in Bangladesh to not continue SANC visits after the first visit resulting in inflation at one perhaps due to cultural trait of not visiting doctors, habit of taking previously prescribed medicine without further consultation with health professional etc. Therefore, to determine the potential factors associated with SANC visits, the ZOIP model has been fitted to this dataset.

## Materials and methods

### Data source

In order to address the objectives of the study, data have been extracted from four recent consecutive Bangladesh Demographic and Health Surveys (BDHS) conducted in 2011, 2014, 2017–18 and 2022 and later combined together to create the final dataset. The data are freely available on request in DHS Program website https://dhsprogram.com/data/available-datasets.cfm. Initially we registered on the Demographic and Health Survey (DHS) Program website and requested to get access to BDHS data mentioning the study objectives. After getting authorization from DHS within two working days, we downloaded and used the women’s dataset from each survey for our study purpose. These surveys gathered up to date information on major maternal and child health related indicators along with socio-economic and demographic factors.

### Study and sample design

The BDHSs are nationally representative cross-sectional surveys. They were conducted using two stage stratified sampling, where the primary sampling units were the enumeration areas considered in the Population and Housing Census of the People’s Republic of Bangladesh prepared by Bangladesh Bureau of Statistics; and the secondary sampling units were a systematic sample of households within the selected enumeration area. Information regarding maternal and child health related indicators was obtained by interviewing ever married women of reproductive age who gave their last birth five years and three years preceding the survey in 2011 and the surveys in 2014, 2017–2018, 2022, respectively. The outline of sample design of the above-mentioned surveys can be found in final reports of the corresponding surveys [4, 20–22].

### Study participants

The BDHS 2011, 2014, 2017–18 and 2022 covered respectively 17,842, 17,863, 20,127 and 30,078 completed interviews of women of reproductive age. In this study, we have considered only women participants who provided information regarding SANC visits for their last birth. We have omitted participants with any missing data i.e. omitted missing value in outcome and explanatory variables and performed analysis on available data only. This process of handing missing data facilitates obtaining unbiased estimates when missing observations in the dataset occur completely at random [23]. Consequently, we selected 6,956, 4,304, 4,819 and 4,712 women from four waves of BDHSs conducted in 2011, 2014, 2017–18 and 2022, respectively and the resulting sample is of size 20,791 for the combined dataset that ensures complete information on all the selected variables in the study.

### Ethical approval and consent to participate

The DHS Program was reviewed and approved by the Institutional Review Boards of the ICF International, Rockville, Maryland, USA as the program complied with all of the requirements of 45 CFR 46, “Protection of Human Subjects”. The Bangladesh DHSs (BDHS) 2011, 2014, 2017–18 and 2022 were classified under that approval. Meanwhile, the National Research Ethics Committee of the Bangladesh Medical Research Council, Dhaka, Bangladesh also provided approval for BDSHs. Mitra and Associates, Dhaka, Bangladesh, a private research agency collected data for these BDHSs under the authority of the National Institute of Population Research and Training (NIPORT) of the Government of the People’s Republic of Bangladesh with the financial support from USAID/Bangladesh. Informed consent was obtained from each survey participant prior to asking questions by reading a written consent in front of the participant and taking signature or thumb print. The participants who refused to give consent were excluded from the surveys. The participants’ identification was kept anonymous and confidential. All methods were carried out according to relevant guidelines and regulations.

### Outcome variable

As the objective of this study is to investigate the trend of SANC practice and its determinants, the number of antenatal care visits of a pregnant woman during her last birth provided by skilled health attendants is considered as the response variable. According to WHO, skilled health attendants are the health providers who are either qualified doctors, nurses, midwives, paramedics, family welfare visitors, or community skilled attendants [24]. According to BDHS, skilled health providers are (1) qualified doctors, (2) nurses/ midwives/ paramedics, (3) family welfare visitors, (4) sub-assistant community medical officers, (5) community skilled birth attendants [4]. In this study, information on ANC visits is considered as SANC if a woman received this service from any of the skilled health professionals.

### Independent variables

On the basis of the literature, a number of socioeconomic and demographic variables have been considered as the independent variables for this study which may influence SANC visits [6, 10–15]. The ‘area of residence’ of the mother has been taken as an independent variable and classified as urban area and rural area. ‘Maternal age at index birth’ has been grouped into three categories: below 20 years, between 20 and 34 years and above 34 years. ‘Maternal education level’ has been taken as a covariate with four categories: no education, primary, secondary and higher. ‘Paternal education level’ has similar categories as the maternal education level. In this study, the ‘wealth index’ of the mother has been categorized into poor, middle and rich. ‘Birth order’ of a child consists of three categories: 1st birth, 2nd or 3rd birth, and above 3rd birth. ‘Mother’s working status’ has two categories: working and not working. Two categories have been used for ‘media exposure’: exposed and non-exposed. The variable ‘participation in decision making’ has been formed with two categories: yes and no. The variable ‘opinion on violence against women’ has been formed with two categories: justified and not justified. The variable ‘wanted pregnancy’, when became pregnant with index birth, consists of two categories: yes and no. The variable ‘ever had terminated pregnancy’ has been classified into two categories: yes and no. The ‘survey year’ has been used as an ordinal variable with four categories: 2011, 2014, 2017–18 and 2022.

It is to be noted that some variables have been constructed by recoding the available variables in the datasets. The wealth index variable has been created from the original variable wealth index (consisting of five categories: poorest, poorer, middle, richer, richest) by merging poorest and poorer into poor category and richer and richest into rich category. For the variable ‘media exposure’, the exposed category has been defined for a mother who reads newspapers or magazines or watches television or listens to radio not less than once a week. One of the indices for women empowerment is mother’s participation either alone or jointly with spouse in household decisions, such as, (i) mother’s own health care, (ii) major household-related purchases and (iii) visiting relatives. Thus, the yes category of the variable ‘participation in decision making’ has been defined if the mothers involve in any of the three decisions. Another index for women empowerment is the opinion of women regarding justification of wife beating for the reasons such as (i) arguing with husband, (ii) neglecting the children, (iii) going out without telling husband, (iv) refusing husband for intercourse and (v) burning the food. The justified category of this variable includes a mother in favor of any of the five reasons of wife beating. Wanted pregnancy, when became pregnant with index birth, consists of three categories in the original data set: then, later, and no more. The categories ‘then’ and ‘later’ have been merged to form the ‘yes’ category of this variable.

### Statistical analyses

In the study, the pooled dataset from the aforementioned BDHSs have been used to explore the pattern of SANC visits among the reproductive women in Bangladesh over time as well as to determine the potential factors associated with the number of SANC visits. All the explanatory variables considered in the study are categorical and the response variable, the number of SANC visits during last pregnancy is discrete in nature. We have conducted univariate analysis using percent distribution for categorical variables and computing mean and standard deviation for discrete response; bivariate analysis using Kruskal Wallis test and regression analysis using Zero and One Inflated Poisson (ZOIP) model for the pooled dataset.

Firstly, for descriptive statistics, we have performed percent distribution of the explanatory variables and computed mean and standard deviation of the response variable for the pooled dataset. Since the response variable is discrete in nature and cannot follow normal distribution, we have conducted the Kruskal-Wallis test, a nonparametric counterpart of one way ANOVA, to draw inference about the unadjusted association between the explanatory variables and the count response [25]. Moreover, if significant association is found for a covariate having more than two categories, Mann-Whitney U test has been performed for multiple comparisons which determines whether there is any significant difference between the central value of any two groups of an independent variable [25].

After that, we have performed a partial score test for simultaneous inflation at zero and one in the absence of covariates to examine whether the pooled dataset contains excess zeros and excess ones [26]. Finally, the ZOIP model has been applied for analyzing the number of SANC visits by a woman during her last pregnancy in Bangladesh. This model is appropriate when the underlying data is zero and one inflated.

### Model

The ZOIP model is based on three subpopulations, one of the subpopulations provides structural zeros following a degenerate distribution at zero with probability *φ*_0_, another subpopulation consists of structural one counts following degenerate distribution at one with probability *φ*_1_ and the other sub-population provides non-negative counts following Poisson distribution with parameter *λ* (> 0). Let a random sample of size *n* be considered and *Y*_*i*_ be the response for the *i*^*th*^ individual having Zero and One Inflated Poisson (ZOIP) distribution [26–28] of the following form

$$P\left(Y_{i}=y_{i}\right)={\left(\varphi_{0}+\varphi_{2}e^{-\lambda_{i}}\right)}^{I\left(y_{i}=0\right)}\times {\left(\varphi_{1}+\varphi_{2}\lambda_{i}e^{-\lambda_{i}}\right)}^{I\left(y_{i}=1\right)}\times {\left(\varphi_{2}\frac{\lambda_{i}^{y_{i}}e^{-\lambda_{i}}}{y_{i}!}\right)}^{I\left(y_{i}>1\right)},$$

where *φ*_2_ = 1 − *φ*_0_ − *φ*_1_ and *I*(.) is an indicator variable, defined as *I*(*y*_*i*_ = *c*) = 1 if *y*_*i*_ = *c*, otherwise 0. To introduce covariates in ZOIP model, one can define *λ*_*i*_ as a function of covariates *x*_*i*_ through a log link function. i.e., $ln\left(\lambda_{i}\right)=x_{i}^{T}\beta$; where *x*_*i*_ and *β* are *p* × 1 vector of covariates and corresponding regression coefficients, respectively. It is to be noted that no covariate has been introduced for parameters *φ*_0_ and *φ*_1_ since the focus of this study is on the mean of count response. The regression parameters are interpreted using mean ratio defined as $\frac{\lambda_{i}|x_{ij}=\left(k+1\right),\tilde{x_{l}}}{\lambda_{i}|x_{ij}=k,\;\tilde{x_{l}}}\;=\text{exp}\;\left(\beta_{j}\right)$, given that there is no interaction term with the quantitative covariate of interest *x*_*j*_, where $\tilde{x_{l}}$ indicates all covariate except *x*_*ij*_. Note that for binary covariate *k* = 0. The parameters of the ZOIP regression model were estimated using the following likelihood function.

$$l\left(\beta ,\varphi_{0},\varphi_{1}|y\right)=A+B+C,$$

where $A=\sum_{y_{i}=0}\;\text{ln}\;\left[\varphi_{0}+\varphi_{2}\;\text{exp}\left(-e^{x_{i}^{T}\beta }\right)\right]$, $B=\sum_{y_{i}=1}\;\text{ln}\;\left[\varphi_{1}+\varphi_{2}e^{x_{i}^{T}\beta }\;\text{exp}\left(-e^{x_{i}^{T}\beta }\right)\right]$, and $C=\sum_{y_{i}>1}\left[{\text{y}}_{\text{i}}x_{i}^{T}\beta -e^{x_{i}^{T}\beta }+ln\varphi_{2}-\text{ln}\left(y_{i}!\right)\right]$.

After carefully selecting initial values, the R ‘optim’ function can be utilized for the purpose of model fitting using the above log-likelihood function.

### Test for simultaneous inflation at zero and one

Zhang et al. developed a partial score test for examining the concurrent existence of ‘extra zero’ and ‘extra one’ in the dataset [26]. The partial score test statistic asymptotically follows Chi-square distribution with (*k* − 1) degrees of freedom, where *k* is the number of parameters.

## Results

### Descriptive statistics

Table 1 demonstrates survey-specific distribution of the response, the number of SANC counts by mothers from Bangladesh during their last pregnancy. A gradual increase in the average SANC counts along with 95% confidence can be observed from 2011 to 2017–18; then a stagnant decline in 2022. For example, in survey year 2011, the mean SANC count was 2.03 with standard deviation (SD) 2.70, whereas, the average was 2.32 (SD 2.57) in 2014, 3.64 (SD 3.05) in 2017–18 and 3.13 (SD 2.36) in survey year 2022.

**Table 1:** Survey year-specific distribution of SANC counts with 95% confidence interval (CI).

Survey Year	Frequency	Mean ± SD	95% CI
**2011**	6956	2.03 ± 2.70	(1.96, 2.09)
**2014**	4304	2.32 ± 2.57	(2.25, 2.40)
**2017–18**	4819	3.64 ± 3.05	(3.56, 3.73)
**2022**	4712	3.13 ± 2.36	(3.06, 3.20)
**Pooled Sample**	20791	2.71 ± 2.77	(2.67, 2.75)

Table 2 represents the percent distribution of the selected demographic and socio-economic variables by survey years. The pooled data considered a total of 20,791 respondents, particularly, 6,956 respondents from BDHS 2011, 4,304 of those from BDHS 2014, 4,819 of those from BDHS 2017–18 and the rest of 4,712 from BDHS 2022.

**Table 2:** Percentage distribution of selected demographic and socio-economic variables for different survey years.

Covariates with categories		Survey Year				Pooled (n = 20791)
		2011 (n = 6956) (%)	2014 (n = 4304) (%)	2017–18 (n = 4819) (%)	2022 (n = 4712) (%)	
**Area of residence**	Urban	31.6	32.2	34.4	32.9	32.7
	Rural	68.4	67.8	65.6	67.1	67.3
**Maternal age at index birth**	<20	25.8	28.0	24.2	19.1	24.3
	20–34	68.5	67.4	71.2	74.7	70.4
	>34	5.7	4.6	4.6	6.2	5.3
**Maternal education level**	No Education	17.8	13.1	6.1	5.2	11.2
	Primary	29.7	27.1	27.5	22.8	27.1
	Secondary	43.7	48.0	47.9	52.6	47.6
	Higher	8.8	11.8	18.4	19.4	14.1
**Paternal education level**	No Education	26.5	22.4	13.7	14.5	19.9
	Primary	28.9	30.0	33.5	29.6	30.4
	Secondary	30.2	31.9	33.1	34.7	32.2
	Higher	14.4	15.7	19.8	21.2	17.5
**Wealth index**	Poor	39.8	39.5	41.6	40.5	40.3
	Middle	19.2	19.2	18.0	19.8	19.1
	Rich	41.0	41.3	40.4	39.7	40.6
**Birth order**	1^st^ birth	33.9	40.7	38.0	37.1	37.0
	2^nd^ or 3^rd^ birth	47.6	45.5	50.0	53.3	49.0
	Above 3^rd^ birth	18.6	13.8	12.0	9.6	14.0
**Mother’s working status**	Working	9.8	21.4	37.3	25.2	22.9
	Non-working	90.2	78.6	62.7	74.8	77.1
**Exposure of media**	Exposed	65.7	62.6	64.2	59.6	62.7
	Non-exposed	34.3	37.4	35.8	40.4	37.3
**Participation in decision making**	Yes	75.1	72.7	85.0	81.6	78.3
	No	24.9	27.3	15.0	18.4	21.7
**Opinion in violence against women**	Justified	32.7	28.2	18.0	11.8	23.2
	Non-justified	67.3	71.8	82.0	88.2	76.8
**Wanted pregnancy**	Yes	86.6	89.9	91.8	92.6	89.9
	No	13.4	10.1	8.2	7.4	10.1
**Ever had terminated pregnancy**	Yes	18.1	14.8	17.0	18.1	17.2
	No	81.9	85.2	83.0	89.9	82.8

It is apparent from Table 2 that the ratio of women from rural to urban remained in close proximity among different surveys, 2011 to 2022, i.e. approximately one-third of the respondents belong to urban areas of Bangladesh. It has been further found that majority of women (70.4%) were from the 20–34 years age group at their index birth in the pooled dataset. A gradual growth in the literacy rate of parents has been observed during the course of time, with those having no education decreasing to about one-third among mothers and about half among fathers. The proportion of mothers from poor families is around 40% in different survey years, while the sample covered the least number (around 19%) of mothers from middle income families. There has been a declining pattern in the percentage of mothers with four or more births from 18.6% (in 2011) to 9.6% (in 2022). Although there has been a major progress in the percentages of working mothers from 9.8% (in 2011) to 25.2% (in 2022), the percentage of the mothers exposed to media clung to around 60% over time. One can witness preponderance in the percentage of mothers taking part in decision making (78.3%), concurrently, a minority of mothers (23.2%) justified their attitude towards violence against women in the pooled dataset. More than four-fifth of overall mothers (89.9%) wanted the index child and overall 17.2% mothers have been found who had pregnancy terminated earlier.

### Bivariate analysis

Table 3 illustrates the mean number of SANC visits by selected covariates for four consecutive survey data and for the combined dataset. Additionally, for investigating the empirical association between the response, SANC visits and selected covariates in the pooled dataset, Kruskal-Wallis test has been performed. In case of significant association for a covariate having more than two categories, non-parametric Mann-Whitney U test has been performed for multiple comparisons.

**Table 3:** Mean number of SANC visits by different demographic and socio-economic variables with p-value of Kruskal-Wallis test and p-value of multiple comparison by Mann- Whitney U test over the survey years.

Covariates with categories		Survey Year				Overall	p-value (Kruskal-Wallis test)	Pair of categories (p-value of Mann Whitney U test)
		2011	2014	2017–18	2022			
**Area of residence**	Urban	3.22	3.24	4.55	3.81	3.68	<0.001	
	Rural	1.47	1.89	3.16	2.80	2.24		
**Maternal age at index birth**	<20 (T)	1.98	2.22	3.55	2.92	2.56	<0.001	T-M(0.013)
								T-O(<0.001)
	20–34 (M)	2.09	2.39	3.70	3.19	2.80		M-O(<0.001)
	>34 (O)	1.43	1.89	3.31	3.10	2.33		
**Maternal education level**	No Education (N)	0.75	0.96	1.82	1.88	1.05	<0.001	N-P(<0.001)
								N-S(<0.001)
								N-H(<0.001)
	Primary (P)	1.18	1.52	2.64	2.21	1.79		P-S(<0.001)
								P-H(<0.001)
	Secondary (S)	2.49	2.62	3.81	3.12	2.98		S-H(<0.001)
	Higher (H)	5.18	4.49	5.31	4.61	4.93		
**Paternal education level**	No Education (N)	0.87	1.20	2.37	2.25	1.42	<0.001	N-P(<0.001)
								N-S(<0.001)
								N-H(<0.001)
	Primary (P)	1.48	1.72	2.88	2.40	2.09		P-S(<0.001)
								P-H(<0.001)
	Secondary (S)	2.42	2.76	3.94	3.27	3.06		S-H(<0.001)
	Higher (H)	4.40	4.20	5.32	4.54	4.64		
**Wealth index**	Poor (P)	0.91	1.29	2.53	2.25	1.68	<0.001	P-M(<0.001)
								P-R(<0.001)
	Middle (M)	1.60	2.04	3.64	2.98	2.46		M-R(<0.001)
	Rich (R)	3.31	3.45	4.48	4.11	3.85		
**Birth order**	1^st^ birth (F)	2.65	2.74	4.18	3.42	3.21	<0.001	F-S(<0.001)
								F-T(<0.001)
	2^nd^ or 3^rd^ birth (S)	2.01	2.29	3.58	3.08	2.70		S-T(<0.001)
	Above 3^rd^ birth (T)	0.94	1.21	2.18	2.32	1.45		
**Mother’s working status**	Working	2.30	2.13	3.46	3.04	2.92	<0.001	
	Non-working	2.00	2.38	3.75	3.17	2.65		
**Exposure of media**	Exposed	2.58	2.95	4.30	3.61	3.28	<0.001	
	Non-exposed	0.96	1.26	2.46	2.50	1.76		
**Participation in decision making**	Yes	2.13	2.44	3.67	3.24	2.84	<0.001	
	No	1.71	2.02	3.49	2.68	2.26		
**Opinion in violence against women**	Justified	1.53	1.84	2.98	2.75	2.00	<0.001	
	Non-justified	2.27	2.51	3.79	3.19	2.93		
**Wanted pregnancy**	Yes	2.16	2.42	2.51	3.18	2.83	<0.001	
	No	1.15	1.46	3.74	2.58	1.70		
**Ever had terminated pregnancy**	Yes	2.21	2.64	3.95	3.56	3.00	<0.001	
	No	1.98	2.27	3.58	3.04	2.66		
**Survey year**	2011 (F)					2.03	<0.001	F-S(<0.001)
								F-T(<0.001)
								F-U(<0.001)
	2014 (S)					2.32		S-T(<0.001)
								S-U(<0.001)
	2017–18 (T)					3.64		T-U(<0.001)
	2022 (U)					3.13		

Table 3 depicts that the mean number of SANC visits taken by women in urban areas and rural areas are 3.68 and 2.24, respectively, in the combined dataset. In accordance with the Kruskal-Wallis test, the variable ‘area of residence’ has a significant association with SANC visits (p-value <0.001). By undergoing the mean SANC visits decomposed by survey years, an increasing pattern in mean SANC visits is apparent in both urban and rural areas of Bangladesh from 2011 to 2017–18, then a gradual decline in 2022. For instance, the mean SANC counts increased from 1.47 (in 2011) to 3.16 (in 2017–18), then decreased to 2.80 (in 2022) in rural areas, while it improved from 3.22 (in 2011) to 4.55 (in 2017–18), then declined to 3.81 (in 2022) in urban areas of Bangladesh. The results from Table 3 also indicate that in pooled dataset, the mean SANC visits (mean 2.80) was significantly (with p-value <0.001) higher among mothers who were 20–34 years old at index birth compared to mothers who were below 20 years (mean 2.56) or 34+ years old (mean 2.33); similar pattern is also perceptible in any survey year. As anticipated, a significantly (p-value <0.001) higher average SANC visits has been observed among women who are highly educated (mean 4.93) than their other counterparts. Analogous results (p-value <0.001) can be seen for the ‘paternal education level’ variable. The results of Table 3 also signify that the rich subpopulations have taken significantly (p-value < 0.001) higher SANC services (with mean 3.85) than other subpopulations. However, a significant reduction (p-value <0.001) in mean SANC visits has been reflected with the increase in birth order. In addition, working women (with mean 2.92) are more likely to take SANC services than non-working women (with mean 2.65) in the combined dataset and this difference is significant (p-value <0.001). The results of Table 3 also remark that women exposed to any type of media, on an average, have more SANC visits (mean 3.28) compared to women not exposed to media (mean 1.76). A mother’s SANC visits have been found to be significantly positively associated with her participation in decision making (p-value <0.001), whereas these have been inversely associated with her opinion on violence against women (p-value <0.001). Moreover, women having a desire for pregnancy (p-value<0.001) have been more susceptible to taking SANC services (mean 2.83) compared to those with unwanted pregnancy (mean 1.70). Women previously facing a terminated pregnancy (p-value <0.001) have been found to have significant association with their average SANC visits. Lastly, the results from Table 3 revealed that the mean number of SANC visits taken by pregnant women significantly (p-value <0.001) increased from 2011 to 2017–18, then reduced in 2022. For instance, the average SANC visits were 2.03, 2.32, 3.64 and 3.13 in 2011, 2014, 2017–18 and 2022, respectively.

### Score test: Simultaneous inflation at zero and one

To examine whether the pooled data contain excess zeros and excess ones, the aforementioned partial score test for simultaneous inflation at zero and one has been conducted. The p-value of the test has been found as less than 0.001. The result indicates that the pooled data simultaneously contain extra zero and extra one counts. Therefore, the Zero and One Inflated Poisson (ZOIP) regression model seems to be a reasonable choice to analyze the dataset.

### Regression analysis

Since the data are Zero and One Inflated, to examine the adjusted association of the selected covariates with the response variable, ZOIP regression model has been fitted to the pooled dataset.

In this study, we have also examined whether the mean number of SANC visits changes over the time by area of residence. To address this issue, an interaction factor between area of residence and survey year has been considered in the model. It is to be noted that survey year has been taken as a quantitative variable in the model for convenience and the values have been assigned to the variable as 1 for 2011, 2 for 2014, 3 for 2017–18 and 4 for 2022.

In this study, the estimates of regression parameters obtained from Poisson regression model and that of nuisance parameters ‘extra-zero proportion’ and ‘extra-one proportion’ from the ZOIP model in the absence of covariates have been used as the ‘initial values’ in R ‘optim’ function for inference purpose. Results obtained from the fitted ZOIP regression model to the pooled dataset have been presented in Table 4. The mean ratio for the main effects area of residence and survey year are 0.593 (p-value <0.001) and 1.035 (p-value <0.001), respectively, while the mean ratio for interaction effect is 1.120 (p-value <0.001).

**Table 4:** Estimate of parameters, their standard error (SE), mean ratio and p-value for different demographic and socio-economic variables obtained from Zero and One Inflated Poisson regression model.

Covariates with categories		Estimate	SE	Mean ratio	p-value
**Intercept**		0.103	0.040	1.108	0.011
**Area of residence**	Urban			1	
	Rural	-0.523	0.024	0.593	<0.001
**Maternal age at index birth**	<20			1	
	20–34	0.032	0.013	1.033	0.015
	>34	0.148	0.026	1.160	<0.001
**Maternal education level**	No Education			1	
	Primary	0.326	0.027	1.385	<0.001
	Secondary	0.490	0.027	1.632	<0.001
	Higher	0.618	0.030	1.855	<0.001
**Paternal education level**	No Education			1	
	Primary	0.079	0.019	1.082	<0.001
	Secondary	0.201	0.019	1.223	<0.001
	Higher	0.316	0.021	1.371	<0.001
**Wealth index**	Poor			1	
	Middle	0.125	0.015	1.133	<0.001
	Rich	0.211	0.014	1.235	<0.001
**Birth order**	1^st^ birth			1	
	2^nd^ or 3^rd^ birth	-0.032	0.011	0.969	0.004
	Above 3^rd^ birth	-0.222	0.023	0.801	<0.001
**Mother’s working status**	Non-working			1	
	Working	0.077	0.011	1.080	<0.001
**Exposure of media**	Non-exposed			1	
	Exposed	0.243	0.012	1.275	<0.001
**Participation in decision making**	No			1	
	Yes	0.087	0.012	1.091	<0.001
**Opinion in violence against women**	Non-justified			1	
	Justified	-0.061	0.013	0.940	<0.001
**Wanted pregnancy**	No			1	
	Yes	0.14	0.021	1.150	<0.001
**Ever had terminated pregnancy**	No			1	
	Yes	0.104	0.012	1.110	<0.001
**Survey year**		0.034	0.006	1.035	<0.001
**Survey year × Area of residence**		0.114	0.018	1.120	<0.001
**Extra zero proportion (*φ***_**0**_ **)**		0.172	0.004	-	<0.001
**Extra one proportion (*φ***_**1**_ **)**		0.011	0.002	-	<0.001

**Fig 1:**
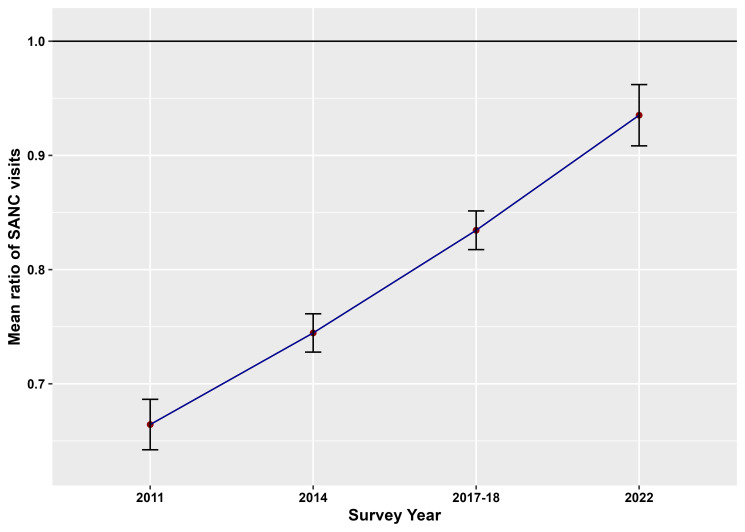
Mean ratio of SANC visits for rural area compared to urban area in different survey years.

**Fig 2:**
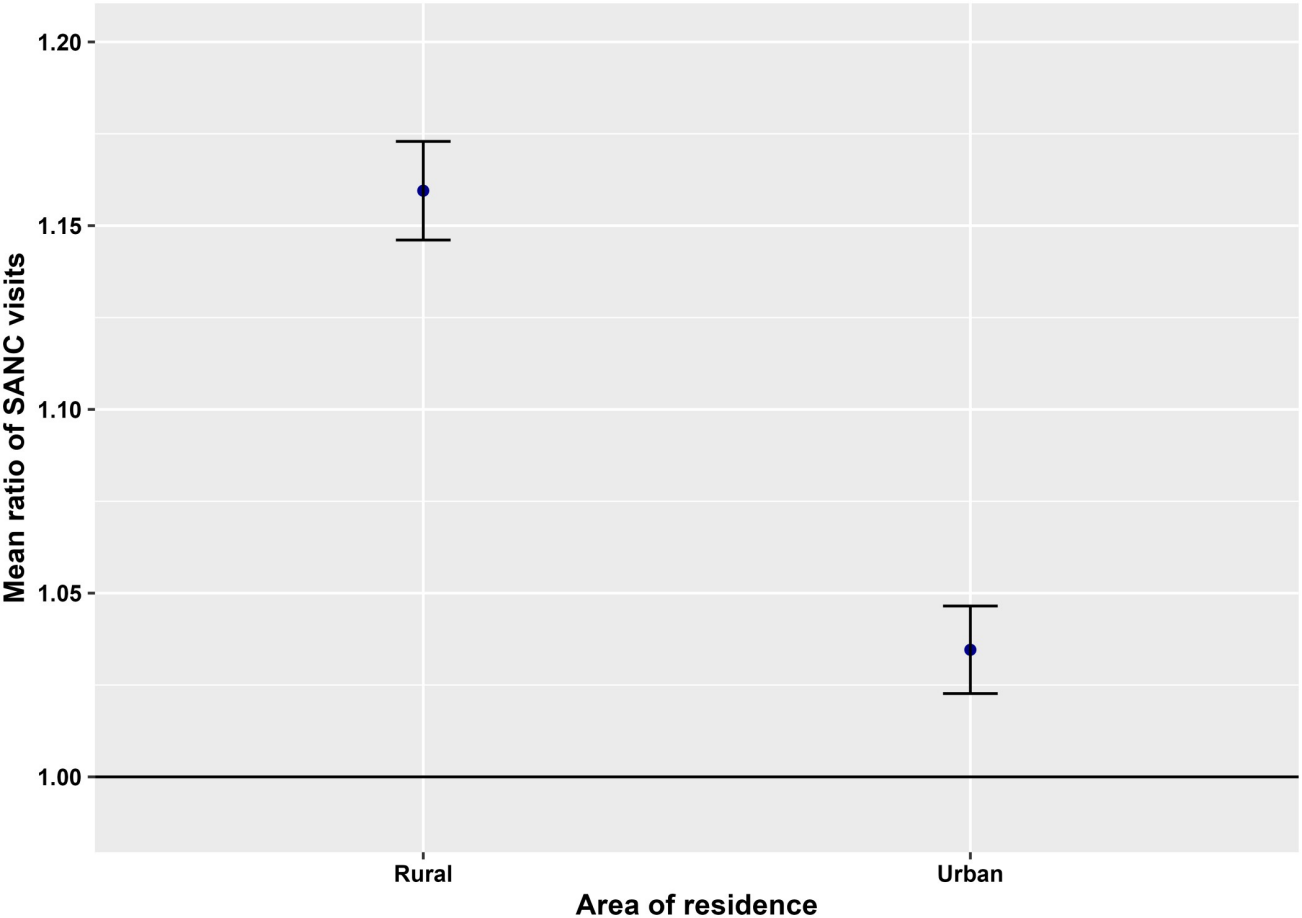
Mean ratio of SANC visits for survey year based on area of residence.

Furthermore, Fig 1 represents the change in the rate of mean SANC visits in rural areas compared to urban areas in different survey years, whereas the change in the rate of mean SANC visits over the time for both rural areas and urban areas has been displayed in Fig 2 with corresponding 95% confidence intervals (CIs). Delta method [29] has been implemented for computing the standard errors of mean ratios in order to formulate the 95% confidence intervals.

From Fig 1, it is apparent that the mean ratios of SANC visits are below 1 for each survey year, which exemplifies that women from rural areas are less likely to take SANC services compared to women from urban areas of Bangladesh. Furthermore, the magnitude of the mean ratio decreases with the survey year indicating the increase in the number of SANC visits by women in rural Bangladesh over time. For instance, the mean number of SANC visits by women in rural areas were 33.6%, 25.6%, 16.6% and 6.5% less compared to that by women in urban areas in Bangladesh in 2011, 2014, 2017–18 and 2022, respectively. While Fig 2 discloses the mean ratios of SANC visits to be above 1 for each area of residence, which demonstrates that the mean number of SANC visits increased with the increase in time in both rural and urban areas of Bangladesh. Furthermore, the change in the rate of taking SANC visits was higher in rural areas compared to that in urban areas in Bangladesh, e.g., for one unit increase in time, the mean number of SANC visits increased by 15.3% in rural areas and 3.5% in urban areas.

It is found from Table 4 that the mean number of SANC visits taken by middle aged mothers (aged 20–34 years) is significantly 1.033 times of that of mothers aged <20 years (p-value 0.015). While the average number of SANC visits by mothers aged 34+ years is significantly 1.160 times of that by teen aged mothers with p-value < 0.001. It is noticed that the increase in parents’ education level results in the increase in the mean number of SANC visits. One can observe a significant positive association between the number of SANC visits and education level of any parent (corresponding p-values <0.001). As for example, considering ‘no education’ as the reference category, the expected number of SANC visits by mothers having primary education, secondary education and higher education are respectively, 38.5%, 63.2%, 85.5% higher than that by mothers having no education. Meanwhile, the average SANC counts by women whose husbands have completed primary education, secondary education and higher education are 1.082 times, 1.223 times, 1.371 times, respectively, of that by women whose husbands are uneducated. Moreover, one can notice that women from poor families are significantly least likely to have SANC services than their other counter parts, as the average SANC visits by mothers from middle class families and rich families are significantly 1.133 times and 1.235 times, respectively, of that by mothers from poor families (corresponding p-values < 0.001). However, reduction in the mean SANC visits is evident with increasing birth order, such as, mothers at their second or third birth and mothers at their above third birth have, on an average, 3.1% and 19.9% lower SANC visits, respectively, in comparison to that by mothers at their first birth; these values are statistically significant as corresponding p-values are 0.004 and < 0.001, respectively. Additionally, working mothers are 8.0% more likely to take SANC services compared to non-working mothers (p-value < 0.001) and the mean SANC visits for the mothers exposed to media is 1.275 times of that of mothers non-exposed to media (p-value is < 0.001). The SANC visits by mothers who take part in decision making are, on an average, 9.1% higher compared to their counterparts (p-value < 0.001). However, the expected number of SANC visits by mothers in favor of violence towards women is 6.0% lower in comparison to that by mothers against it and the result is statistically significant as p-value is < 0.001. Furthermore, mothers with intended last pregnancy are more inclined to take SANC services and their mean SANC count is 1.150 times of that by mothers with unintended last pregnancy (p-value is < 0.001). Meanwhile, the average SANC visits by mothers who ever had terminated pregnancy is 1.110 times of that by mothers who never had such experience (p-value < 0.001). In addition, this model provides evidence of 17.2% extra zero counts and 1.1% extra one counts, with corresponding p-values <0.001.

## Discussion

In different research disciplines, one may often encounter count data having a large frequency of zeros than expected from Poisson distribution. Accordingly, there are substantial number of literatures regarding different mixture models to handle such data. However, in practice, besides excess zero counts, one may witness accumulation of excess one counts in the dataset; for which the models proposed for zero-inflated data may provide inconsistent and less precise results. The Zero and One Inflated Poisson (ZOIP) distribution is more suitable in such situations to overcome the problem of simultaneous inflation at zero and one in Poisson count data.

The objective of this study is to identify potential factors associated with number of skilled antenatal care (SANC) visits and determine the pattern of SANC visits over time in Bangladesh. For this purpose, data extracted from four consecutive BDHSs have been combined and found to be simultaneously zero and one inflated. Therefore, the ZOIP model has been utilized to meet the study objective.

According to our study, a gradual increase in the mean SANC visits from 2011 to 2017–18 followed by a slight decline in 2022 can be observed. In 2022 survey data, we have considered pregnant women who had their last births three years preceding the survey, covering the period from 2020 to 2022 and their interviews were conducted from June to December 2022. The decline in the mean SANC visits in 2022 may have resulted from the adverse effects of COVID-19. Lockdowns during the pandemic at different intervals put a barrier to access healthcare facilities including closure of many hospitals and health clinics, scarcity of doctors in clinics for treating other than covid cases, restrictions in hospitals for regular visits. Along with that, restrictions on movement, unavailability of public transport, financial difficulties due to loss of jobs, increased risk and fear of infection during pregnancy may have discouraged pregnant women to seek in-person antenatal care services during that crisis period [30–32].

The study results also depict that women in urban areas are more accustomed to receiving SANC services compared to women in rural areas of Bangladesh. Inequity in SANC services based on area of residence prevails, one of the reasons being the absence of ample proficient health providers in rural areas because mostly skilled doctors prefer to get posted in urban areas of Bangladesh in search of better facilities [33]. Additionally, women from rural household, mostly residing under the poverty line, have less access to maternal health services because of unavailability of hospitals or clinics in nearby areas, scarcity of female health staff in clinics, hindrance to take health related decision, progressive mindset of household head, indifference to visit doctors due to lack of education and so on [34–36]. However, the gap in the rate of mean SANC visits in rural areas compared to urban areas has gradually reduced over time. The study further highlights that the rate of mean SANC visits is rapidly rising with time among pregnant women not only in the urban areas but also in the rural areas of Bangladesh. In fact, for a given survey year, the rate was higher in urban areas compared to that in rural areas. The findings lay down the fact that Bangladesh has made subtle progress in securing SANC services for pregnant women over time, yet a massive progression is required in this regard to meet the WHO recommendation. Thus, it is of utmost need to address the demographic and socio-economic factors influencing the increase in SANC visits to meet the WHO recommendation and SDGs.

Moreover, this study reports that the variation in mothers’ age at index birth exerts influence on their frequency of SANC visits, in particular, teen mothers have more tendency to skip SANC visits compared to their counterparts. Haque et al. obtained analogous results in the context of Bangladesh [37]; meanwhile, a study in Guinea by Ahinkorah et al. and another one in Ethiopia by Tsegaye et al. obtained similar results [38, 39]. Teen mothers being unmindful about the significance of timely SANC visits or having unintended pregnancy at that age may lead to their low uptake of SANC services [40]. However, Kabir and Islam found the odds of four plus ANC visits least among mothers of aged 35 years or more at conception [41]. Besides, Hossain et al. found that the mean ANC visits was less among mothers of age 20 years or less as well as mothers of age 35+ years compared to that among mothers aged 20–35 years at the time of delivery [14].

Furthermore, the study discloses a significant correlation between mothers’ attainment of education and their likelihood of SANC attendance. This finding is in harmony with some previous studies [14, 15, 37, 42, 43]. Formal education can bring about a change in mothers’ perception towards skilled antenatal care seeking behavior by enhancing their knowledge on health issues, exposing them to health promoting ideas [38, 43, 44], evolving their capacity to make constructive decisions regarding their own health [38, 45–47] which may eventually result in greater utilization of SANC service.

Concurrently, it is prominent from this study that husbands’ education attainment can put a major impact on their wives’ SANC service utilization. In a patriarchal society set-up in Bangladesh, husbands having formal education may address their wives’ healthcare needs during pregnancy and make constructive healthcare choices on their behalf [48, 49], such as taking wife for SANC visits on time, consulting with health professional. Some previous studies [12, 43, 47] are in support of our study finding, however, finding of Tsegaye et al. conflicts with ours [39].

A strong positive association between mothers’ wealth index and their propensity of SANC service utilization can be identified in this study, which matches with previous evidence [14, 15, 37, 42, 43]. One plausible reason may be the fact that in Bangladesh where one third of the general mass live under the poverty line [33], socio-economic status certainly impacts their living standard and molds women’s health related choices in due course [12]. Women from poor households are more reluctant to have SANC service due to the cost of medication, transportation, accommodation etc. when place is far from home. [39, 50]. Alternatively, women from affluent families may have easier exposure to any sort of media that enlighten them about health care necessities as well as they may have more autonomy regarding their health-related decisions compared to women from underprivileged families [39, 43, 51].

The study elucidates the fact that working women are more receptive to antenatal care service from skilled providers compared to non-working women. This is because financial independence gives them more competence to take health related decisions, subsequently lead to more SANC counts. Nonetheless, the finding contradicts with Khan et al. [11].

The study further reveals that exposure to media can play an indispensable role in mothers’ greater uptake of SANC services by bringing their attention to pregnancy related complications through different programs, promoting the necessity of maintaining a healthy lifestyle for safeguarding the unborn child [12], hence stimulating the idea of having more SANC visits. The result is consistent with some previous studies [11–15].

The study sheds light on the fact that higher birth order results in the reduction of SANC visits by pregnant women of Bangladesh. Uncertainties or excitement of the first child may make parents more anxious during the first pregnancy [12], on the other hand, having previous experience of live birth may illuminate the anxiety. The study result is in favor of the findings of some previous studies [6, 12, 14, 15, 41].

The study further reveals that women with intended pregnancy are more susceptible to receiving SANC services. Several previously conducted studies came up with similar findings [12, 52–54]. Women willing to have children are often more cautious about receiving antenatal care services from skilled providers timely out of the fear of any pregnancy complications or miscarriage. Alternatively, women with untimely or unintended pregnancy are found less concerned about SANC visits, Ahinkorah et al. even stated that women with unintended pregnancy may embrace the idea of abortion, which alternatively reduces their ANC counts [38, 55].

Apart from that, women who previously experienced terminated pregnancy showed adherence to receiving SANC services compared to women with no such experience. Unpleasant pregnancy experience prior to the current pregnancy may give a rise to their SANC counts [56, 57]. Studies conducted in low-middle income countries (LMIC) also notified similar outcomes [56, 58].

This study reflects on two indices of women empowerment, which are: women’s participation in decision making and their opinion on violence towards women. Both indices have been found to improve the frequency of SANC visits by pregnant women in Bangladesh. Similar to our results, Bhowmik et al. and Ousman et al. identified women’s decision-making capacity regarding their health care as a significant cause of rise in their ANC counts [38, 43, 59]. Meanwhile, Kabir and Islam found an increase in odds of taking four plus ANC visits when women made decisions regarding their health care jointly with their husbands [41]. Haque et al. concluded that the inflation in the number of reasons of justifying wife beating was correlated with lower uptake of ANC service, although they obtained no correlation between the number of ANC counts by pregnant women and the number of decisions regarding themselves [37]. Besides, Hossain et al. did not find any association between women empowerment and frequency of ANC counts [14].

WHO recommends receiving the first SANC visit within 12 weeks of conception with additional seven visits throughout the gestational period. Starting from a woman’s conception to child’s second birthday, ensuring optimal health and nutrition for both mother and child is crucial [60]. Essential screenings, preventive measures like tetanus immunization and iron-folic acid supplementation, advising on early breast feeding and health education during SANC visits can improve maternal nutrition, birth preparedness, and overall fetal health. Previous study findings revealed that women receiving ANC in the first trimester were more likely to consume IFA tablets for at least 90 days compared to those who initiated ANC in the second or third trimester [61]. The increasing number of SANC visits had also been found to be associated with higher IFA tablets consumption [62, 63]. Along with that, the number of SANC visits and breastfeeding advice during SANC were positively associated with early initiation of breastfeeding. Precisely, women with four or more visits were more likely to initiate breast feeding within the first hour after birth which strengthens the immune system and supports healthy development of newborn [63].

Lower SANC attendance is also linked to higher preterm birth risk, a major contributor to under-five mortality worldwide [64, 65]. Preterm birth can disrupt early development and lead to long-term health challenges, including learning disabilities, hearing problems, sensory deficits, respiratory illnesses, and a heightened risk of chronic conditions in adulthood. A cohort study in rural Bangladesh revealed women attending three or more ANC visits had significantly lower risk of preterm birth compared to those with one or no visits [65]. Another study conducted in Bangladesh showcased that women with less than four ANC visits were more likely to have a preterm birth compared to those with four or more visits; underscoring the importance of ANC attendance in mitigating preterm births rate [66]. Essential knowledge on neonatal disease prevention, immunization, nutrition, and health practices provided during skilled antenatal care attendance also resulted in reduced neonatal and under-5 mortality improving child health outcomes [67, 68].

Moreover, SANC visits allow healthcare providers to monitor maternal health and fetal development closely and identify and treat potential health issues early, which contribute to healthier birth weights and lower risk of stunting [69–71]. Our study results (in S1 Table) also demonstrate a significant negative association between the number of SANC visits and adverse child health outcomes; that is, the mothers having small increase in SANC visits (e.g. from 2 to 3 visits) had lower odds of giving birth to children being stunted, underweight and wasted (with and without controlling for other covariates). Precisely, pregnant women who had WHO recommended at least 8 SANC visits had significantly 23.2%, 27.4% and 15.1% lower odds of giving birth to a child being stunted, underweight, wasted, respectively, compared to women who had just 2 SANC visits; the results align with previous studies [69, 70].

In a nutshell, the study puts emphasis on the advancement in the number of skilled antenatal care visits by pregnant women in Bangladesh over time disintegrating area of residence. As a matter of fact, the improvement took place with a higher rate in rural areas compared to urban areas of Bangladesh. The study further showcased some of the demographic and socio-economic factors catalyzing the progression.

### Limitations and future scope

To carry out the study purpose, we extracted cross-sectional data from four waves of nationally representative BDHSs. Since BDHS does not include randomized controlled trials (RCTs), the study precludes any causal relationship between the response, number of SANC visits and the covariates. Hence, the nature of the survey also restricts causal inference between the number of SANC visits and adverse child health outcomes. Other than that, the study may possess recall bias due to being retrospective in nature. However, we tried to alleviate the bias to some extent by considering respondents having index birth in the last five years prior to BDHS 2011 and those having index birth in the last three years prior to BDHS 2014, BDHS 2017–18 and BDHS 2022. Additionally, we could not explore association between timing of each antenatal visit and mentioned independent variables since there is no record of timing of each antenatal care visits in BDHS dataset.

As the study revolves around exploring the advancement in the number of skilled antenatal care visits by pregnant women in Bangladesh over time, we did not delve into assessing how the increased skilled antenatal care visits may benefit the pregnant women and newborn. Besides that, investigation of the advantages of skilled antenatal care visits will require more detailed analysis of important health indicators such as, maternal health status, fetal development, birth outcome, child health outcomes, long-term health effects. In future studies, one may explore how the rising number of skilled antenatal care visits by pregnant women in Bangladesh leverages maternal and child health status by incorporating sufficient information of maternal and child health indicators and relevant risk factors as well as including more robust methodologies such as causal inference methods to establish causal relationship between the variables.

## Conclusion

Despite a massive progression in SANC counts over time, Bangladesh still stands far from accomplishing Sustainable Development Goals (3.1) and (3.2), let alone ensuring WHO recommended eight plus skilled antenatal care visits by all pregnant women in the country. Therefore, basic SANC services must be ensured and more health campaigns and health programs should be conducted specially in the rural area of Bangladesh. Maternal health interventions should target teen aged mothers and encourage them to visit the skilled providers during pregnancy. Both male and female education should be promoted effectively to enhance SANC visits and effective steps should be imposed to increase both male and female education level in the country. Necessary actions must be taken so that women from poor families get easy access to SANC services. Additional attention must be paid in persuading mothers to take SANC services during pregnancy of higher order birth just like the first birth. As women who get easy access to media are more likely to seek SANC visits, mass-media campaigns should be strengthened to motivate women to form the habit of reading magazines or newspapers, listening to radios, or watching shows on television regarding maternal and child health. Women’s freedom of working outside their home and participating in decision making should be appreciated with a view to confirming more ANC visits from skilled professionals during pregnancy. Awareness should be created among women with unintended pregnancy about the necessity of taking SANC services during pregnancy and they should be convinced to seek SANC visits. Hence, policy makers should develop appropriate strategies needed for a sustainable increase in the number of SANC visits in Bangladesh by adopting the aforementioned recommendations.

## Supporting information

S1 Table Unadjusted odds ratio (UOR) and adjusted odds ratio (AOR) of different child health outcomes for number of skilled antenatal care (SANC) visits with 95% confidence interval (CI) and p-value using binary logistic regression models. (DOCX)
