# Supplementary material for: Rural-urban disparity in uptaking skilled antenatal care visits by pregnant women in Bangladesh: Zero and One Inflated Poisson regression model
Source: PLoS One. 2025 Jan 29;20(1):e0318341. doi: 10.1371/journal.pone.0318341 (PMC11778761; doi:10.1371/journal.pone.0318341)
Supplement: S1 Table — (DOCX) [file pone.0318341.s001.docx]

**S1 Table: Unadjusted odds ratio (UOR) and adjusted odds ratio (AOR) of different child health outcomes for number of skilled antenatal care (SANC) visits with 95% confidence interval (CI) and p-value using binary logistic regression models.**

| **Stunting** | | | | | | |
| --- | --- | --- | --- | --- | --- | --- |
| Survey year | UOR | 95% CI of UOR | p-value of UOR | AOR | 95% CI of AOR | p-value of AOR |
| 2011 | 0.884 | (0.865,0.903) | <0.001 | 0.958 | (0.935,0.981) | <0.001 |
| 2014 | 0.870 | (0.844,0.897) | <0.001 | 0.935 | (0.905,0.967) | <0.001 |
| 2017-18 | 0.912 | (0.891,0.933) | <0.001 | 0.964 | (0.941,0.988) | 0.004 |
| 2022 | 0.891 | (0.845,0.938) | <0.001 | 0.957 | (0.905,1.011) | 0.119 |
| pooled data | 0.881 | (0.870,0.893) | <0.001 | 0.957 | (0.943,0.971) | <0.001 |
| **Underweight** | | | | | | |
| 2011 | 0.858 | (0.838,0.880) | <0.001 | 0.945 | (0.921,0.969) | <0.001 |
| 2014 | 0.859 | (0.831,0.887) | <0.001 | 0.936 | (0.904,0.969) | <0.001 |
| 2017-18 | 0.913 | (0.888,0.938) | <0.001 | 0.962 | (0.935,0.990) | 0.007 |
| 2022 | 0.861 | (0.814,0.910) | <0.001 | 0.930 | (0.876,0.987) | 0.017 |
| pooled data | 0.853 | (0.840,0.867) | <0.001 | 0.948 | (0.933,0.963) | <0.001 |
| **Wasting** | | | | | | |
| 2011 | 0.932 | (0.906,0.960) | <0.001 | 0.971 | (0.939,1.003) | 0.077 |
| 2014 | 0.927 | (0.892,0.963) | <0.001 | 0.963 | (0.923,1.004) | 0.078 |
| 2017-18 | 0.979 | (0.943,1.016) | 0.257 | 1.000 | (0.962,1.040) | 0.988 |
| 2022 | 0.925 | (0.870,0.983) | 0.012 | 0.927 | (0.865,0.994) | 0.034 |
| pooled data | 0.919 | (0.902,0.936) | <0.001 | 0.973 | (0.953,0.993) | 0.008 |

Note: AOR is obtained by controlling for covariates such as, area of residence, maternal age at index birth, maternal education level, paternal education level, wealth index, birth order, mother’s working status, exposure of media, participation in decision making, opinion in violence against women, wanted pregnancy, ever had terminated pregnancy, survey year.
